# Interleukin-2 gene methylation levels and interleukin-2 levels associated with environmental exposure as risk biomarkers for preterm birth

**DOI:** 10.3325/cmj.2023.64.320

**Published:** 2023-10

**Authors:** Aleksandra Fučić, Jelena Knežević, Jure Krasić, Denis Polančec, Nino Sinčić, Nada Sindičić Dessardo, Mirta Starčević, Vedrana Guszak, Marcello Ceppi, Marco Bruzzone

**Affiliations:** 1 Institute for Medical Research and Occupational Health, Zagreb, Croatia; 2 Srebrnjak Children's Hospital, Zagreb, Croatia; 3 Scientific Center of Excellence for Reproductive and Regenerative Medicine (CERRM), University of Zagreb School of Medicine, Zagreb, Croatia; 4 Biomedical Research Center Šalata, University of Zagreb School of Medicine, Zagreb, Croatia; 5 University Hospital Center Zagreb, Zagreb, Croatia; 6 IRCCS Ospedale Policlinico San Martino, Genova, Italy; The first two authors contributed equally.

## Abstract

**Aim:**

To compare interleukin-2 levels (IL-2) and IL-2 gene site 1 methylation levels between preterm newborns (PN) and full-term newborns (FN) and investigate their association with the environmental exposure of their mothers during pregnancy.

**Methods:**

IL-2 and IL-2 gene site 1 methylation levels were assessed in 50 PN and 56 FN. Newborns’ mothers filled in questionnaires about their living and occupational environments, habits, diets, and hobbies.

**Results:**

The mothers of PN were significantly more frequently agrarian/rural residents than the mothers of FN. PN had significantly higher IL-2 levels, and significantly lower methylation of IL-2 gene site 1 levels than FN.

**Conclusion:**

IL-2 levels, hypomethylation of the IL-2 gene site 1, and the mother’s rural residence (probably due to pesticide exposure) were predictive biomarkers for preterm birth. For the first time, we present the reference values for the methylation of IL-2 gene site 1 in PN and FN, which can be used in the clinical setting and biomonitoring.

Preterm birth (PB) represents a significant medical and social problem. Annually, 15 million newborns worldwide are born preterm (1). The causes of preterm birth range from inflammation, severe preeclampsia, and premature rupture of the membranes to genetic predisposition (2,3). However, preterm birth may also be the consequence of the mother’s environmental exposure, diet, and habits (4,5). The environmental effects that have been reported to affect the duration of the gestational period are increased levels of organochlorine pesticides, air pollution, β-hexachlorobenzene, hexachlorocyclohexane, and heavy metals (6,7). Studies on this topic have been conducted using a limited number of biomarkers.

Preterm newborns have a higher risk of morbidity than FN, which is why they need long-term follow-up and medical monitoring (8-10). Late PN have a higher rate of neurodevelopmental problems and hypertension, with a higher risk of infant death in their offspring (8,11).

Because health risks in PN are frequently associated with immunological and developmental disturbances (12,13), it is important to identify biomarkers that may predict specific health risks. Previous studies revealed that immune immaturity in PN (14) was associated with a higher risk of asthma, bronchiolitis, cardiovascular disturbances, neoplastic diseases, and a weaker response to vaccination (15-20).

Interleukin-2 (IL-2), primarily produced by activated CD4^+^ T cells, drives T cell proliferation and differentiation (21,22). Full-term newborns’ T lymphocytes secrete little or no IL-2, compared with adult T lymphocytes (23,24). Increased IL-2 was shown to be associated with an increased risk of asthma in children, while animal models showed that IL-2 also affected other tissues, such as during cardiovascular recovery (25). In PN, IL-2 levels have been measured rarely, with contradictory results, and in a small number of subjects. The published results showed lower or higher levels of IL-2 in PN compared with FN (26-28).

Many CpG sites have been associated with preterm birth and gestational age (29-31). Differences in CpG site methylation levels were found between preterm and term-birth children at 18 years of age (32). Umbilical cord blood cells from PN differ in DNA methylation levels compared with those of FN, and these differentially methylated sites are involved in different pathways, among others in immune response (33). In addition, methylation in newborns may be significantly modified by the mother’s diet and environmental exposure (34-38).

Although methylation of the IL-2 gene site 1 has been associated with asthma risk in newborns (39), studies investigating DNA methylation disturbances in PN are still limited, while epigenetic biomarkers are not even considered in clinical diagnostics (40,41). The interaction between environmental exposure, methylation, preterm birth risk, and health risks during adulthood has not been well investigated. Prenatal exposure to pesticides was shown not only to significantly affect the immunological system but also to increase the risk of autism. Furthermore, it was associated with deviations in the level of IL-2, which is known to be involved in central nervous system development and normal brain physiology (42,43). Additionally, it has been reported that autism can be characterized by disturbances in DNA methylation (44).

The aim of this study was to: a) measure IL-2 levels in late PN and FN newborns, b) compare the levels of IL-2 gene site 1 methylation in PN and FN, and c) evaluate the association between IL-2 and methylation of IL-2 gene site 1 levels in PN and FN levels with environmental exposure, habits, and diet of their mothers during pregnancy.

## Participants and methods

### Participants

IL-2 levels and IL-2 site methylation levels were assessed in the cord blood of 56 FN and 50 PN and these results were compared with data collected through questionnaires filled out by their mothers. Preterm birth was defined as birth before 37 completed weeks of gestation (GA). In this study, the range of GA weeks in PN was from 27 until 36 weeks, while in the case of FN, the range was from 37 to 41. Only spontaneous preterm birth newborns were included. Newborns too small for gestation age and those with malformations were excluded. The mothers signed written consent and filled in detailed questionnaires about their medical and family history, occupational exposures, diet (meat, soft beverages, alcohol, dairy products, vegetables, fruit), hobbies (use of plastic materials, paint, glue), residence, in-house environment during pregnancy (renovation), and smoking. The exclusion criteria were parental occupational exposure to chemical agents or radiation, parental chemotherapy or radiotherapy during life, parental addiction to drugs, and alcohol abuse. The questionnaire was based on the experiences from the NewGeneris project (45), adjusted for Croatian lifestyle specificities. Cord blood vein samples were collected within a 10-month period. The samples were centrifuged for serum separation for 10 min at 3000 rpm and were frozen at -80 °C. The study was approved by the Ethics Committee of Zagreb University Hospital Center.

### Interleukin-2 measurements

The serum IL-2 concentration was measured with ELISA (Human IL-2 ELISA Kit High Sensitivity, Abcam, Catalog Number #ab46054, Cambridge, UK) according to the manufacturer’s instructions. Serum samples and standards of known IL-2 concentrations were added to the appropriate microplate wells, coated with a monoclonal antibody specific for human IL-2, and simultaneously incubated with a biotinylated monoclonal antibody specific for IL-2 at room temperature. After the washing step, the enzyme streptavidin-HRP that binds to biotinylated antibody was added to the wells and incubated at room temperature. Following another washing step, Chromogen TMB Substrate solution was added, acting on the bound enzyme to induce a colored reaction product. After 15 minutes, the color development, which was directly proportional to the IL-2 concentration present in the samples, was stopped with an appropriate stop reagent. The absorbance was read on a microplate reader, using 450 nm as the primary wavelength and 610 nm as the reference wavelength.

### DNA methylation analysis

Whole blood (500 μL) was mixed with red blood cell lysis buffer (900 μL, 0.32 M Sucrose, 5 mM MgCl2, 1% Triton X-100 and 10 mM Tris-HCl pH 8.0) and centrifuged at 7000 rpm for 10 minutes to purify white blood cell nuclei. A total of 500 μL of nucleic lysis buffer (10 mM EDTA pH 8.0, 10 mM Tris-HCl pH 8.0, 1% SDS and 0.01 mM sodium citrate dihydrate) and 20 μL Proteinase K (20 mg/mL) were added to the white blood cell nuclei pellet and incubated on a thermal shaker at 56 °C and 600 rpm overnight. DNA was then purified and precipitated with a modified salting-out method (Miller, 1988). Finally, DNA was resuspended in 50 μL of TE buffer (pH 8.0). DNA concentration and quality were measured with the NanoDrop ND-2000 spectrophotometer (NanoDrop Technologies, Wilmington, DE, USA). Samples were then stored at -20 °C until further use.

All of the procedures were performed according to the manufacturer’s instructions. In total, 500 ng of isolated genomic DNA was used for bisulfite conversion with an EpiTect Plus DNA Bisulfite Kit (#59124; Qiagen, Hilden, Germany). Then, 10 ng of bisulfite-treated DNA was used as a template for polymerase chain reaction (PCR) amplification of the promoter region of interest with a PyroMark PCR Kit (#978703; Qiagen). Primers and annealing temperatures for PCR and DNA methylation analysis of IL2 were as described by Curtin et al (39). The biotinylated PCR product was purified with a Pyromark Q24 Vacuum Workstation (Qiagen). IL2 promoter methylation levels were then measured with Pyromark Q24 Advanced System with the PyroMark Q24 CpG Advanced Reagents (#970922; Qiagen). DNA methylation levels were calculated as the ratio of C/T at a CpG site with the Pyromark Q24 Advanced Software 3.0.1 (#9022779, Qiagen).

### Statistical analysis

The univariate comparison between controls and pre-term births was performed with a Mann-Whitney test for continuous variables and a Fisher exact test for categorical variables. The multivariate analysis was performed with the generalized linear model (GLM) with a binomial family and logarithmic link to the dichotomous variable (0/1) that labels controls and pre-term births, in order to estimate the risk ratio (RR) (46). The variables that were significantly associated with the type of birth in the univariate analysis and those selected by a stepwise procedure, together with IL2 and methylated IL2, were included in the statistical models. The confidence intervals were estimated with a bootstraping procedure with 1000 replicates. The analysis was performed with Stata Software version 16.1 (StataCorp LLC, College Station, TX, USA).

## Results

IL-2 levels and IL-2 gene site 1 methylation level were assessed in 56 FN (37-42 weeks GA) and 50 PN (27-36 weeks GA). There was no significant difference in sex between PN and FN. PN belonged to the group of late PN according to gestational age (median 35 GW) (Table 1).

**Table 1:** Characteristics of preterm and full-term newborns

	Preterm newborns				Full-term newborns				
	N	Mean, median (SD)	25°/75°*	range	N	Mean, median (SD)	25°/75°	range	*P* value
Gestational age	50	34.10, 35.0 ±2.28	33.0/36.0	27-36	56	39.54, 40 ±1.06	39/40	37-42	<0.001
Weight	50	2284.50, 2350.0 ±614.70	1900.0/2665.0	930-3640	56	3508.20, 3499 ±404.38	3260/3795	2710-4610	<0.001
	**N**	**%**			**N**	**%**			
Sex									0.701
female	23	46.0			28	50.0			
male	27	54.0			28	50.0			
Mother’s age	50	32.10, 33.0 ±6.29	27.0-35.0	20-49	56	31.91.9 ±5.17	29/35	17-42	0.899
Mother’s smoking status	45	90.0			47	83.9			0.402
non-smoker									
smoker	5	10.0			9	16.1			

*25/75 percentile.

In both groups, the mothers did not drink alcohol during pregnancy. There was no significant difference between PN and FN in smoking status, probably due to the very few mothers who smoked during pregnancy. Significantly more mothers who gave birth to PN had an agrarian and rural residence than mothers who gave birth to FN (Table 2).

**Table 2:** Comparison of living environment between the mothers of preterm and full-term newborns during pregnancy

		**Preterm**		Full-term	
	N	%	N	%	*P* value
**Residence**					0.006
Urban	16	32.0	35	62.5	
Rural	7	14.0	5	8.9	
Agrarian	27	54.0	16	28.6	

The Mann-Whitney test showed that the mean value of IL-2 levels was significantly higher in PN than in FN, who did not express IL-2. Methylation of IL-2 gene site 1 levels was significantly higher in FN than in PN (Table 3). Sex differences were not detected in either of the groups with regard to IL-2 levels and IL-2 mehylation gene site levels.

**Table 3:** Interleukin (IL-2) levels and the methylation levels of IL-2 gene

	Preterm				Full-term				
	N	Mean, Median (SD)	25°/75°*	Range	N	Mean, Median (SD)	25°/75°	Range	*P* value
IL-2 MET	47	67.32, 68.0 (5.63)	63.0/71.0	53-80	55	72.76, 73 (4.57)	69/76	61-82	<0.001
IL-2	38	2.10, 0 (4.25)	0-1.65	0-16.78	54	0.00, 0 (0.00)	0/0	0-0	<0.001

*25/75 percentile.

In the multivariate GLM model, the IL-2 levels were dichotomized with respect to zero, while IL-2 gene methylation was dichotomized with respect to the median value of 70.5. The results showed that, when IL2 was greater than zero, the risk of preterm birth was 3.47 (Table 4) and when IL-2 gene site 1 methylation was greater than its median value, the risk of preterm birth was 0.48. A rural residence increased the risk of preterm birth by two times (Table 5), a finding confirming the results of the univariate analysis.

**Table 4:** A generalized linear model for the association between interleukin-2 (IL-2) and preterm birth, adjusted by residence and painting

	Risk ratio	95% confidence interval	*P* value
**IL-2**			
zero	1.00		
nonzero	3.47	1.97-6.10	<0.001
**Residence**			
urban	1.00		
rural	1.41	0.16-12.62	0.757
agrarian	1.61	0.84-3.06	0.150

**Table 5:** A generalized linear model for the association between methylated interleukin 2 (IL-2) gene site 1 and preterm birth, adjusted by residence and painting

	Risk ratio	95% confidence interval	*P* value
**IL-2met**			
53-70.4	1.00		
70.5-82	0.48	0.30-0.76	0.002
**Residence**			
rural	2.10	1.15-3.82	0.016
agrarian	1.84	1.13-2.98	0.014

## Discussion

This is the first study to show that increased levels of IL-2 and decreased IL-2 gene methylation site 1 levels in cord blood were significant predictive factors of preterm birth risk. Other studies also detected higher levels of IL-2 in PN compared with FN (26,28,47,48). IL-2 and IL-2 gene methylation site 1 levels were not associated with the mother’s diet, smoking, coffee consumption, or living habits. Our study is the first to report the levels of IL-2 gene site 1 methylation in PN and FN, which may be used as a reference in future studies and clinical settings, as well as an important biomarker of preterm birth.

The importance of preterm birth prevention is reflected in the United Nations Sustainable Development Goal 3 target #3.2, which aims at avoiding all preventable deaths of newborns and children under 5 years of age by 2030. Due to increased health risks during lifetime and immunological disturbances (43,49), PN require specific biomonitoring and a personalized approach.

Earlier studies demonstrated an association between preterm birth and rural residence (50-52). Also, large-scale studies in humans are increasingly being undertaken to assess the effect of bendiocarb, a carbamate insecticide used in public health and agriculture, on the neonatal immune system (53,54). Bendiocarb was found to cause increased IL-2 in cord blood, and to be correlated with the maternal plasma concentration of bendiocarb (55). In an animal model, organophosphorus insecticides such as pirimiphos-methyl (O-2-diethylamino-6-methylpyrimidin-4-yl O,O-dimethyl phosphorothioate) or endosulfan significantly increased IL-2 production (56,57).

Immunological disturbances such as deviations in cytokine levels in newborns and early childhood may be related to allergies, susceptibility to inflammation, and neurodevelopmental disturbances later in life (39,58,59). Increased IL-2 was shown to obstruct T follicular helper cell differentiation, a process critical for long-term immunity and reinfection (60).

Besides immunological effects, IL-2 has been associated with neurological disturbances during development. Animal models suggested that IL-2 was required for cell development in the mesolimbic and mesostriatal systems, whose pathology is associated with autism and cognitive disturbances (61-63). Autism and deviations in cognitive capacity are also characterized by disturbed IL-2 and associated to pesticide exposure (53,64,65). Thus, our results may contribute to retrospective and prospective research on the associations between transplacental exposure to pesticides, IL-2 levels, and neurodevelopmental risks.

DNA methylation status was shown to be significant in T-cell differentiation during intrauterine development (40). Preterm and term neonates show differences in methylation in umbilical cord T-cells and erythrocytes. Compared with preterm neonates, term neonates have global hypermethylation in term T-cells (3,66-68). The common profile of all preterm newborns is that they carry lifelong patterns of disturbed DNA methylation (3,30). Hypermethylation of IL-2 gene site 1 was observed in newborns of mothers with atopic asthma, while increased methylation indicated an increased risk for asthma exacerbations (39). Hypomethylation of the *IL-2* gene was also found in children with peanut allergy, a finding that could explain their elevated levels of IL-2 when exposed to peanut proteins (69). Although there was no association of spontaneous preterm birth with IL-2 methylation in the African-American population of the US, an association was established with hypomethylation of CYTIP, which is known to be upregulated by IL-2 (70).

Prenatal exposure to fine particles, perfluorinated alkyl compounds, PAHs, and some xenoestrogens has been associated with DNA hypomethylation of leukocytes in the umbilical cord (35,71,72). A single study evaluated mothers’ exposure to dichlorodiphenyltrichloroethane in two small groups of six newborns, who showed disturbances in genome-wide methylation levels (25). Some of the identified pesticides caused hypomethylation in adults (73,74), which suggests that the hypomethylation detected in our study may also be associated with mothers’ exposure to pesticides.

A limitation of this study is the relatively small sample size, which may limit the generalizability of the findings to a broader population.

In conclusion, research on the causes of preterm birth and preventive measures should focus more on transplacental exposure, which may be modified by educating parents through consulting during pregnancy. Increased levels of IL-2 and decreased levels of IL-2 gene site 1 methylation are predictors of preterm birth and may affect health risks during life, associated not only with the immunological system, but also with the central nervous system. The exposure to pesticides should be further investigated, and education about the associated risks should be included in counseling protocols for pregnant women. Further studies should assess disturbances of the IL-2 receptor in PN, as an important part of the involved pathways.
